# Supplementary material for: Effect of Intake of Extra Virgin Olive Oil on Mortality in a South Italian Cohort with and without NAFLD
Source: Nutrients. 2023 Oct 29;15(21):4593. doi: 10.3390/nu15214593 (PMC10649270; doi:10.3390/nu15214593)
Supplement: Supplementary file 1 [file nutrients-15-04593-s001.zip › nutrients-2671097-supplementary.pdf]

Supplementary Materials

Table S1. Daily consumption for food groups, consumption of oil, alcohol and wine by age group (years).

	Age Classes (years)					
	<40	40-49	50-59	60-69	70-79	≥80
	Mean (SD)	Mean (SD)	Mean (SD)	Mean (SD)	Mean (SD)	Mean (SD)
N (%)	601 (21.8)	565 (20.5)	540 (19.6)	505 (18.3)	424 (15.4)	119 (4.3)
Fruits (gr/die)	626.36 (527.10)	748.34 (608.91)	687.06 (562.04)	669.24 (530.04)	666.81 (560.00)	538.01 (408.12)
Vegetables (gr/die)	250.47 (198.65)	248.63 (229.02)	246.05 (182.71)	265.84 (223.81)	252.23 (191.86)	174.10 (114.14)
Legumes (gr/die)	34.72 (30.27)	31.13 (23.98)	33.29 (24.94)	35.38 (43.32)	33.08 (25.49)	28.07 (21.72)
Cereals (gr/die)	295.61 (144.20)	287.92 (143.45)	278.72 (145.54)	248.41 (132.34)	238.06 (125.73)	229.46 (106.83)
Fish (gr/die)	38.40 (29.65)	39.04 (30.20)	40.43 (29.18)	37.56 (38.06)	32.77 (28.31)	28.71 (24.55)
EVO oil (gr/die)	25.95 (16.31)	26.76 (14.90)	35.70 (17.74)	42.32 (21.08)	44.43 (19.14)	39.16 (17.35)
Total Meat (gr/die)	97.74 (57.98)	88.11 (49.22)	83.05 (53.69)	68.02 (44.94)	59.96 (38.63)	54.41 (47.49)
Dairy Products (gr/die)	229.53 (155.74)	217.55 (159.76)	189.73 (154.29)	183.07 (118.85)	204.01 (139.33)	245.39 (184.58)
Alcohol (ml/die)	139.66 (205.60)	167.75 (245.62)	219.53 (274.47)	212.61 (273.30)	163.87 (217.23)	107.91 (117.82)
Wine (ml/die)	94.59 (158.20)	130.71 (194.96)	174.94 (217.00)	176.73 (221.48)	145.35 (186.49)	103.79 (112.61)
Kcalories days	2274.68 (718.58)	2266.59 (717.92)	2255.51 (730.77)	2143.86 (706.28)	2083.12 (672.89)	1920.17 (637.51)

Fruit: Peaches, Watermelon, Apricots, Cherries, Grapes, White Melon (Spring and Summer) Apples, Pears, Citrus Fruits and Kiwis (Autumn and Winter). Vegetables: Aubergines, Courgettes, Spinach, Cabbage, Cauliflower, Turnip Greens, Cucumbers, Watermelons, Green Beans, Fava Beans with Vegetables, Carrots, Artichokes, Peppers, Fennel, Celery, Swiss Chard, Chicory, Green Salad. Salad Tomatoes. Legumes: Broad Beans, Chickpeas, Beans, Lentils and Peas. Cereals: Barley, Pasta, Rice, Pizza, Focaccia, Bread, Biscuits, Pastries. Fish: Octopus, Cuttlefish, Squid, Shrimps, Anchovies, Sardines, Mussels, Mullet, Mackerel. Total Meat: Veal, Lamb, Horse, Rabbit, Chicken, Liver, Pork, Sausage, Mortadella, Ham, Salami. Dairy products: Milk, Yoghurt, Matured Cheese, Fresh Cheese, Mozzarella, Ricotta.

Table S2. Daily consumption for food groups, consumption of oil, alcohol and wine by rMED Score.

	rMED		
	Low (0-6)	Medium (7-10)	High (11-18)
	Mean (SD)	Mean (SD)	Mean (SD)
N (%)	N=634	N=1.664	N=347
Fruits (gr/die)	509.65 (490.49)	708.12 (562.75)	805.95 (555.35)
Vegetables (gr/die)	169.62 (184.32)	256.03 (187.12)	370.08 (254.86)
Legumes (gr/die)	22.60 (17.22)	34.40 (25.89)	48.45 (53.44)
Cereals (gr/die)	280.78 (149.38)	272.29 (138.52)	247.20 (124.51)
Fish (gr/die)	31.50 (24.59)	37.88 (32.00)	46.31 (34.63)
EVO oil (gr/die)	28.72 (18.20)	35.36 (19.34)	38.98 (18.54)
Total Meat (gr/die)	104.30 (59.66)	76.61 (47.81)	54.04 (34.99)
Dairy Products (gr/die)	275.82 (165.71)	200.88 (135.96)	117.60 (111.99)
Alcohol (ml/die)	230.81 (275.60)	170.12 (238.28)	96.48 (138.14)
Wine (ml/die)	168.45 (217.59)	139.90 (195.63)	86.89 (126.14)
Kcalories days	17.53 (22.64)	14.56 (20.36)	9.04 (13.13)

Fruit: Peaches, Watermelon, Apricots, Cherries, Grapes, White Melon (Spring and Summer) Apples, Pears, Citrus Fruits and Kiwis (Autumn and Winter). Vegetables: Aubergines, Courgettes, Spinach, Cabbage, Cauliflower, Turnip Greens, Cucumbers, Watermelons, Green Beans, Fava Beans with Vegetables, Carrots, Artichokes, Peppers, Fennel, Celery, Swiss Chard, Chicory, Green Salad. Salad Tomatoes. Legumes: Broad Beans, Chickpeas, Beans, Lentils and Peas. Cereals: Barley, Pasta, Rice, Pizza, Focaccia, Bread, Biscuits, Pastries. Fish: Octopus, Cuttlefish, Squid, Shrimps, Anchovies, Sardines, Mussels, Mullet, Mackerel. Total Meat: Veal, Lamb, Horse, Rabbit, Chicken, Liver, Pork, Sausage, Mortadella, Ham, Salami. Dairy products: Milk, Yoghurt, Matured Cheese, Fresh Cheese, Mozzarella, Ricotta.
